# Tackling inequalities in obesity: a protocol for a systematic review of the effectiveness of public health interventions at reducing socioeconomic inequalities in obesity amongst children

**DOI:** 10.1186/2046-4053-1-16

**Published:** 2012-02-23

**Authors:** Clare L Bambra, Frances C Hillier, Helen J Moore, Carolyn D Summerbell

**Affiliations:** 1 Department of Geography, Wolfson Research Institute, Durham University Queen's Campus, University Boulevard, Stockton-on-Tees, TS17 6BH, UK; 2 Obesity Related Behaviours Research Group, School of Medicine and Health, Wolfson Research Institute, Durham University Queen's Campus, University Boulevard, Stockton-on-Tees, TS17 6BH, UK

## Abstract

**Background:**

There is growing evidence of the impact of overweight and obesity on short- and long-term functioning, health and well-being. Internationally, childhood obesity rates continue to rise in some countries (for example, Mexico, India, China and Canada), although there is emerging evidence of a slowing of this increase or a plateauing in some age groups. In most European countries, the United States and Australia, however, socioeconomic inequalities in relation to obesity and risk factors for obesity are widening. Addressing inequalities in obesity, therefore, has a very high profile on the public health and health services agendas. However, there is a lack of accessible policy-ready evidence on what works in terms of interventions to reduce inequalities in obesity.

**Methods and design:**

This article describes the protocol for a National Health Service Trust (NHS) National Institute for Health Research-funded systematic review of public health interventions at the individual, community and societal levels which might reduce socioeconomic inequalities in relation to obesity amongst children ages 0 to 18 years. The studies will be selected only if (1) they included a primary outcome that is a proxy for body fatness and (2) examined differential effects with regard to socioeconomic status (education, income, occupation, social class, deprivation and poverty) or the intervention was targeted specifically at disadvantaged groups (for example, children of the unemployed, lone parents, low income and so on) or at people who live in deprived areas. A rigorous and inclusive international literature search will be conducted for randomised and nonrandomised controlled trials, prospective and retrospective cohort studies (with and/or without control groups) and prospective repeat cross-sectional studies (with and/or without control groups). The following electronic databases will be searched: MEDLINE, Embase, CINAHL, PsycINFO, Social Science Citation Index, ASSIA, IBSS, Sociological Abstracts and the NHS Economic Evaluation Database. Database searches will be supplemented with website and grey literature searches. No studies will be excluded on the basis of language, country of origin or publication date. Study inclusion, data extraction and quality appraisal will be conducted by two reviewers. Meta-analysis and narrative synthesis will be conducted. The main analysis will examine the effects of (1) individual, (2) community and (3) societal level public health interventions on socioeconomic inequalities in childhood obesity. Interventions will be characterised by their level of action and their approach to tackling inequalities. Contextual information on how such public health interventions are organised, implemented and delivered will also be examined.

**Discussion:**

In this review, we consider public health strategies which reduce and prevent inequalities in the prevalence of childhood obesity, highlight any gaps in the evidence base and seek to establish how such public health interventions are organised, implemented and delivered.

PROSPERO registration number: CRD42011001740

## Background

There is growing evidence of the impact of overweight and obesity on short- and long-term functioning, health and well-being [1]. Internationally, childhood obesity rates continue to rise in some countries (for example, Mexico, India, China and Canada), although there is emerging evidence of a slowing of this increase or a plateauing in some age groups. In most European countries, the United States and Australia [2,3], however, socioeconomic inequalities in relation to obesity and risk factors for obesity are widening [1,4-7]. Obesity is causally linked to chronic diseases such as diabetes, coronary heart disease, stroke, hypertension, osteoarthritis and certain forms of cancer [8]. It is predicted that as the UK population grows and ages, the burden of diseases associated with obesity will cost the National Health Service Trust (NHS) £10 billion per year by 2050 [4] and will result in escalating numbers of early deaths as well as long-term incapacity and associated reductions in quality of life [8]. Childhood obesity is a particular concern, and it is widely accepted that there is a link between childhood obesity and morbidity and mortality in later life [9,10]. Tackling obesity is therefore rightly highlighted as one of the major contemporary public health policy challenges and vital in terms of addressing health inequalities [4,8]. The Foresight review of obesity also highlighted the importance of taking a whole-systems approach to tackling the 'obesity epidemic' [4], whereby interventions target the broader societal determinants of obesity [5].

### Inequalities in obesity

In the United Kingdom, like other high-income countries, obesity is associated with social and economic deprivation, with a higher prevalence in the lowest income quintile [11]. Current research suggests that this gradient is embedded, with little evidence of change over time [10]. Geographical inequalities are also evident, with hot spots in the Northeast, Yorkshire and Humber, as well as in the East and West Midlands [10]. The social patterning of obesity in adults is mirrored in children also, with children of low socioeconomic status having higher rates of obesity [7,11]. Data derived from longitudinal analyses suggest that social disadvantage accumulated throughout the life course has an impact on widening inequalities in relation to obesity in adulthood and that this trend is particularly marked amongst women [11].

### Policy context

Addressing inequalities in obesity has a very high profile on the public health agenda in the United Kingdom and internationally. However, there is a lack of accessible policy-ready evidence on what works in terms of interventions to reduce inequalities in relation to obesity. Existing systematic reviews have examined only the effects of interventions which reduce overall levels of obesity, as opposed to the effects on inequalities in relation to obesity. There is, therefore, no information to help policy-makers and commissioners of services assess the types of interventions that are most effective at reducing inequalities in relation to obesity. This evidence gap has been noted in the recent report of the Priority Public Health Conditions group (Task Group 8) of the Department of Health-commissioned Strategic Review of Health Inequalities in England Post 2010 (Marmot Review) [12,13], in which an overt call was made for evidence syntheses on the types of interventions that work to reduce inequalities in obesity prevalence, how they work and under which circumstances they work. The Evidence for Policy and Practice Information and Co-ordinating Centre (EPPI-Centre) report on childhood obesity also called for future systematic reviews to examine the effectiveness of interventions in reducing inequalities and improving the obesity levels of disadvantaged groups [14] (p. 41). Similarly, at the international level, Robertson *et al*. identified the need for "evidence of the reach and penetration of interventions in lower income groups" as a priority area for research [6] (p. 140). The review also has international relevance, given the importance attached to "the development and testing of social determinants of health indicators and intervention impact evaluation" by the World Health Organisation (WHO) Commission on the Social Determinants of Health [15] (p. 23). It is critical for policy-making in this area that evidence of the effectiveness of different types of interventions at tackling inequalities is systematically identified, appraised and synthesised.

Furthermore, there is increasing recognition amongst policy-makers that to tackle complex health problems such as obesity effectively and to reduce health inequalities require integrated policy action across different intervention levels (individual, community and societal), as well as across the life course (childhood to adulthood) [4,11]. The organisation and implementation of such interventions are also important [16]. Against this backdrop, the systematic review proposed herein will address this deficit in the knowledge base by reviewing primary studies of the effectiveness of interventions to reduce inequalities in relation to obesity in a whole-systems way. The review will therefore examine public health interventions at the individual, community and societal levels [17]. It will also examine the organisation, implementation and delivery of interventions.

### Intervention framework

**Figure 1:**
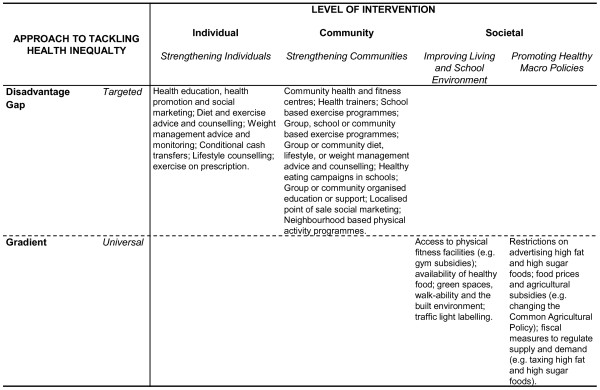
**A framework for tackling inequalities in obesity**.

We have developed a framework for how inequalities in relation to obesity might be tackled (Figure 1). This framework shows that interventions are characterised by their level of action and their approach to tackling inequalities. Following Whitehead [18], there are four levels of interventions that can be used to tackle inequalities: strengthening individuals (person-based strategies to improve the health of disadvantaged individuals), strengthening communities (improving the health of disadvantaged communities and local areas by building social cohesion and mutual support), improving living and school environments (reducing exposure to health-damaging material and psychosocial environments across the whole population) and promoting healthy macroscopic policies (improving the macroeconomic, cultural and environmental contexts which influence the standard of living of the whole population). According to Graham and Kelly, these interventions are underpinned by one of three different approaches to health inequality: disadvantage (improving the absolute position of the most disadvantaged individuals and groups), gap (reducing the relative gap between the best- and worst-off groups) or gradient (reducing the entire social gradient) [19]. Interventions are thus either targeted (such as individual-level interventions which are underpinned by health as a disadvantage) or universal (such as interventions based on living and school conditions which potentially influence the entire social gradient in health). In the proposed systematic review, the obesity interventions will be grouped according to this framework (with acknowledgement that some interventions such as Sure Start might be cross-cutting [18]). For example, as Figure 1 shows, exercise and diet advice is a targeted intervention aimed at strengthening individuals or communities in disadvantaged circumstances and underpinned by a disadvantage approach to health inequality.

Within the framework, a number of different types of interventional components exist (Table 1). Although specific interventional components will tend to cluster within certain categories in the framework, for example societal level interventions tend to involve regulation- and taxation-type components, some types will exist within more than one framework category, and a framework category may contain more than one type of interventional component. An intervention can also contain a number of different elements; for example, a school-based obesity prevention programme may involve educational (healthy eating advice), regulatory (school meal standards) and subsidy (free after-school sport classes) components.

**Table 1:** Intervention component typology

Intervention component	Examples
Education	Food labelling, healthy lifestyle advice, counselling
Regulation	Advertising, retail location, greenbelts
Taxation	Fat ingredient tax, sugar ingredient tax
Subsidy	Free public parks, gardens, play areas, school-based sport and exercise classes
Incentive	Payment for weight loss, reward points for eating healthy school dinners, conditional cash transfer

## Methods and design

The review will be carried out following established criteria for the good conduct and reporting of systematic reviews [20,21]. A Study Steering Group comprising key stakeholders from the UK policy and research communities, international representatives, a statistician and a health economist will guide the research. The review is registered with the PROSPERO International Prospective Register of Systematic Reviews (registration number CRD42011001740).

### Objectives

This project has two objectives: (1) to systematically review the effectiveness of public health interventions (individual, community and societal) in reducing socioeconomic inequalities in obesity amongst children and (2) to establish how such public health interventions are organised, implemented and delivered.

### Interventions

The review will examine public health interventions at the individual, community and societal levels which might reduce inequalities in relation to obesity amongst children ages 0 to 18 years (including prenatal) in any setting and in any country. The review will utilise the intervention framework (Figure 1) and group interventions by intervention component typology (Table 1). Where possible, the obesity interventions will be grouped according to these types with the acknowledgement that some interventions might be cross-cutting. The review will consider public health strategies which might reduce existing inequalities in the prevalence of obesity as well as those interventions that might prevent the development of inequalities in relation to obesity. However, clinical interventions such as those involving drugs or surgery and laboratory-based studies will be excluded.

### Study designs

A rigorous and inclusive international literature search will be conducted for all randomised and nonrandomised controlled trials, prospective and retrospective cohort studies (with and/or without control groups) and prospective repeat cross-sectional studies (with and/or without control groups) of the effectiveness of public health interventions at reducing inequalities in relation to childhood obesity. Studies with a duration of at least 12 weeks (combination of intervention and follow-up) will be included, a criterion used in previous Cochrane reviews of interventions aimed at preventing obesity in children [22] and of the effectiveness of exercise for weight loss in adults with overweight or obesity [23].

### Search strategy

The search strategy will include the following electronic database searches (host sites given in parentheses): MEDLINE (Ovid), Embase (Ovid), CINAHL (NHS Evidence Health Information Resources), PsycINFO (NHS Evidence Health Information Resources), Social Science Citation Index (Thomson Reuters Web of Science), ASSIA (CSA), IBSS (EBSCO Publishing, Barnet, UK), Sociological Abstracts (CSA) and the NHS Economic Evaluation Database (NHS CRD). The skills of a trained information scientist (HJM) will be used to develop and implement the electronic searches (see Additional file 1 for MEDLINE search strategy). All databases will be searched from start date to the present. We will not exclude papers on the basis of language, country of origin or publication date.

We will supplement the electronic database searches with website and grey literature searches. We will hand-search the bibliographies of all included studies and request relevant information on unpublished and in-progress research from key experts in the field. In addition, we will hand-search the last two years of the five most common journals revealed by the electronic searches as well as journals identified by experts in the subject area. We will also contact study authors for unpublished data on health inequalities.

### Outcomes

In terms of outcomes, we will only include studies if they include a primary outcome that is a proxy for body fatness (weight and height, body mass index, waist measurement, waist-to-hip proportion, percentage body fat content, skin fold thickness and ponderal index in relation to childhood obesity). Data on related secondary outcomes (such as physical activity levels, dietary intake and blood test results such as cholesterol and glucose levels) will also be extracted from those studies which have a primary outcome. We will include both measured and self-reported outcomes. Studies will be included only if the researchers examined differential effects with regard to socioeconomic status (education, income, occupation, social class, deprivation and/or poverty) or the intervention was targeted specifically at disadvantaged groups (for example, children of the unemployed, lone parents, low-income families, and so on) or individuals living in deprived areas. Data on the organisation, implementation and delivery of interventions will be extracted using existing methodological tools which assess the implementation of complex public health interventions [16] adapted and refined for the purposes of this review. Examples of the implementation components that will be examined include theoretical underpinning, implementation context, experience level of the intervention team (planners and implementers), consultation and/or collaboration processes (planning and delivery stages) and resources (for example, time, money, staff and equipment).

### Data extraction and quality appraisal

The initial screening of titles and abstracts will be conducted by one reviewer (FCH), with a random 10% of the sample checked by a second reviewer (HJM). Full-paper study inclusion and data extraction will be conducted by two reviewers (FCH and HJM) independently using established data extraction forms [20,24-29]. Any discrepancies will be resolved through discussion between the authors and, if consensus is not reached, with the project lead (CLB). The methodological quality of the included studies will also be appraised independently by two reviewers (FCH and HJM) using the Cochrane Public Health Review Group's recommended Effective Public Health Practice Project Quality Assessment Tool for Quantitative Studies [30], which includes, amongst other things, an examination of sampling strategy, response and follow-up rates, intervention integrity, statistical analyses and assessment of adjustment for confounders. We will use the quality appraisal criteria for descriptive purposes and to highlight variations between studies.

### Analysis and synthesis

Where possible, meta-analysis will be used to synthesise data using Comprehensive Meta-Analysis software (Biostat, Englewood, NJ, USA) based on the mean differences derived from the primary outcomes. A fixed-effect model will be used for the meta-analysis unless there is evidence of heterogeneity between studies, in which case a random-effect model will be considered. The presence of heterogeneity will be investigated with the use of a likelihood ratio test statistic, whilst funnel plots will be considered to explore publication bias. Where meta-analysis is not possible, however, narrative synthesis will be conducted. We will report our analyses in accordance with the PRISMA guidelines [31]. The main analysis will examine the effects of (1) individual, (2) community and (3) societal level public health interventions on socioeconomic inequalities in relation to obesity using the multidimensional framework outlined in Figure 1 and the typology of intervention components given in Table 1. We will examine differential effectiveness by socioeconomic status. Interventions will also be grouped according to the age group targeted: prenatal, early years and primary and secondary school-age interventions (as well as generic all-age interventions). Where data permit, we will conduct demographic subgroup analysis by age, gender and ethnicity.

## Discussion

The review will consider public health strategies which reduce existing inequalities in the prevalence of obesity as well as those interventions that might prevent the development of inequalities in obesity. The review will also serve as a mapping exercise of the types of interventions that have been evaluated in relation to tackling inequalities in relation to obesity amongst children, thereby highlighting any gaps in the evidence base. The review will also seek to establish how public health interventions which might reduce or prevent inequalities in obesity are organised, implemented and delivered. Context is increasingly recognised as an important factor in the success of public health interventions [17]. However, the assessment of implementation has not really featured strongly in previous obesity reviews. We will therefore develop, refine and apply existing methodological tools which assess the implementation of complex public health interventions [16].

The study design inclusion criteria in the review are broad, given that whilst trials of individual, and even community, level interventions are likely, we expect a dearth of experimental studies in relation to societal level interventions. Indeed, large evaluations, such as those of Change4Life (England) and Ensemble, Prévenons l'Obésité des Enfants (EPODE; France), have all used a repeat cross-sectional design. This is perhaps because, as Law and colleagues observed [11], societal level interventions tend not to be easily evaluated using experimental study designs. Furthermore, other recent systematic reviews of the effects of societal level public health interventions on socioeconomic inequalities in relation to health have located few relevant experimental studies [32].

We anticipate that our extensive search strategy, combined with the inclusive study design criteria, will ensure that a sizeable literature will be located for synthesis. Recent Cochrane Heart Group reviews of interventions that prevent and treat obesity amongst children found 22 and 64 randomised controlled trials, respectively [22,33]. Whilst we acknowledge that the literature on the effects of interventions on health inequalities is likely to be smaller, we will maximise the likelihood of locating relevant studies by taking a more inclusive approach to study design, contact study authors for unpublished data on health inequalities, and evaluate interventions targeted at deprived groups or areas as well as studies that include comparative data on the effects of interventions on differential impacts across two or more socioeconomic groups. The size of the available evidence base will also be extended, because we will look at different levels of intervention: individual, community and societal. We will also examine the full papers of all studies which fit our population, intervention, design and health outcome inclusion criteria, even if there is no mention of socioeconomic inequalities in the abstract. By adopting this strategy, we will be less likely to exclude studies which undertook subgroup analyses by socioeconomic status but did not publish the findings in the abstract. We will then contact the study authors for possible subgroup analyses and request any additional unpublished data on health inequalities. This will increase the comprehensiveness of the search strategy and therefore the quality of the final synthesis.

Once the evidence has been synthesised, an 'implications for policy and practice' review dissemination workshop will be held with invited NHS commissioners whose responsibilities include obesity, Department of Health policy-makers with responsibilities for obesity and inequalities, user group representatives (for example, community groups, schools and employer organisations, trade union congresses), as well as UK research network representatives (for example, the Faculty of Public Health, Nutrition Society and the United Kingdom Public Health Association) to discuss the results, aid in the write-up and facilitate the translation of the findings into practice. The technical report and executive summary will then be finalised, and a short 'key findings' summary of the research will be sent to relevant stakeholders. The research will be disseminated via national and international academic and/or practitioner cross-over conferences, and a policy-orientated summary paper will be published on an open access basis so that it is freely available to practitioners and the public.

## Competing interests

The authors declare that they have no competing interests.

## Authors' contributions

CLB is the principal investigator of this project and led the writing of the manuscript. FCH is the project manager and co-investigator of the project and contributed to the writing and revision of the manuscript. HJM and CDS are co-investigators of this project and contributed to the writing and revision of the manuscript. All authors read and approved the final manuscript.

## Supplementary Material

Additional file 1 **MEDLINE (Ovid) Search Strategy: 1946 to 10 October 2011**. The search terms used in the MEDLINE (Ovid) electronic bibliographic database. Click here for file
