# Supplementary material for: Tackling inequalities in obesity: a protocol for a systematic review of the effectiveness of public health interventions at reducing socioeconomic inequalities in obesity amongst children
Source: Syst Rev. 2012 Feb 23;1:16. doi: 10.1186/2046-4053-1-16 (PMC3351709; doi:10.1186/2046-4053-1-16)
Supplement: Additional file 1 — MEDLINE (Ovid) Search Strategy: 1946 to 10 October 2011. The search terms used in the MEDLINE (Ovid) electronic bibliographic database. [file 2046-4053-1-16-S1.DOC]

**Search Strategy – MEDLINE (Ovid) 1946 to 10 October 2011**

1. "Body Weights and Measures"/
2. (BMI or Body Mass Index).ti,ab. or Body Weight/ or obesity.ti,ab. or obese.ti,ab. or overweight.ti,ab. or weight gain.ti,ab. or weight loss.ti,ab. or exp OBESITY/ or Body fat.ti,ab. or Fat mass.ti,ab. or Weight control$.ti,ab. or Weight maintain$.ti,ab. or Adipos$.ti,ab. or Adipose tissue.ti,ab. or Skinfold thickness.ti,ab. or Waist circumference.ti,ab. or Waist hip ratio.ti,ab. or WHR.ti,ab.
3. 1 or 2
4. Health Promotion/ or health promotion.ti,ab. or health behaviour.ti,ab. or health behavior.ti,ab. or (policy and (social or school or food or public or urban or environmental or fiscal)).ti,ab. or urban planning.ti,ab. or city planning.ti,ab. or built environment.ti,ab. or social environment.ti,ab. or physical environment.ti,ab. or cultural environment.ti,ab. or urban environment.ti,ab. or school environment.ti,ab. or neighbourhood.ti,ab. or community.ti,ab. or societal.ti,ab. or social interventions.ti,ab. o! r community interventions.ti,ab. or obesogenic environment.ti,ab. or i ndividual level.ti,ab. or lifestyle.ti,ab. or individual.ti,ab. or tax$.ti,ab. or subsid$.ti,ab. or price$.ti,ab. or health education.ti,ab. or social marketing.ti,ab. or (diet and (advice or counselling)).ti,ab. or (exercise and (advice or counselling)).ti,ab. or weight management.ti,ab. or cash transfer$.ti,ab. or lifestyle counselling.ti,ab. or behavioural counselling.ti,ab. or behavioral counselling.ti,ab. or exercise on prescription.ti,ab. or exercise.ti,ab. or health trainer$.ti,ab. or school.ti,ab. or workplace.ti,ab. or campaign$.ti,ab. or (access adj1 facilities).ti,ab. or green space.ti,ab. or walk?ability.ti,ab. or food label$.ti,ab. or food advert$.ti,ab.
5. (evaluat$ or effective$ or Intervention or RCT or experiment$ or randomi?ed controlled trial$ or clinical randomi?ed controlled trial$ or cluster randomi?ed controlled trial$ or double blind randomi?ed controlled trial$ or randomi?ed consent design or single blind randomi?ed controlled trial$ or randomi?ed or placebo or random$ or trial or quasi?experiment$ or pre$test or post$test or trial or time series or evaluat$ or intervention$ or "before and after" or intervention$ or commun! ity trial or non?randomi?ed or repeat$ or repeat$ measures).ti,ab. or (exp Clinical Trial/ or exp Randomized Controlled Trial/ or exp Randomization/ or exp Double-Blind Method/ or exp Single-Blind Method/ or exp Cross-Over Studies/) or clinical trial.ti,ab. or latin square.ti,ab. or random$.ti,ab. or exp Evaluation/ or clinical trial.ti,ab. or clinical trial.pt. or (before adj1 after adj1 (stud$ or trial$ or design$)).ti,ab. or random$.ti,ab. or (quasi?experimental or pseudo?experimental).ti,ab. or (nonrandomi?ed or non?randomi?ed or pseudo?randomi?sed or quasi?randomi?ed).ti,ab. or ((population level or population based or population orientated or population oriented or community level or community based or community orientated or community oriented) adj3 (intervention$ or prevention or policy or policies or program$ or project$)).ti,ab.
6. 3 and 4 and 5
7. limit 6 to humans
